# GRACE satellite observations reveal the severity of recent water over-consumption in the United States

**DOI:** 10.1038/s41598-017-07450-y

**Published:** 2017-08-18

**Authors:** Kurt C. Solander, John T. Reager, Yoshihide Wada, James S. Famiglietti, Richard S. Middleton

**Affiliations:** 1 Los Alamos National Laboratory, Los Alamos, NM 87545 USA; 2 Jet Propulsion Laboratory, California Institute of Technology, 4800 Oak Grove Drive, Pasadena, California 91009 USA; 3 International Institute for Applied Systems Analysis, Laxenburg, Austria; 4 Center for Climate Systems Research/NASA Goddard Institute for Space Studies, Columbia University, 2880 Broadway, New York, New York, 10025 USA; 5 Department of Physical Geography, Utrecht University, Utrecht, The Netherlands

## Abstract

Changes in the climate and population growth will critically impact the future supply and demand of water, leading to large uncertainties for sustainable resource management. In the absence of on-the-ground measurements to provide spatially continuous, high-resolution information on water supplies, satellite observations can provide essential insight. Here, we develop a technique using observations from the Gravity Recovery and Climate Experiment (GRACE) satellite to evaluate the sustainability of surface water and groundwater use over the continental United States. We determine the annual total water availability for 2003–2015 using the annual variability in GRACE-derived total water storage for 18 major watersheds. The long-term sustainable water quantity available to humans is calculated by subtracting an annual estimate of the water needed to maintain local ecosystems, and the resulting water volumes are compared to reported consumptive water use to determine a sustainability fraction. We find over-consumption is highest in the southwest US, where increasing stress trends were observed in all five basins and annual consumptive use exceeded 100% availability twice in the Lower Colorado basin during 2003–2015. By providing a coarse-scale evaluation of sustainable water use from satellite and ground observations, the established framework serves as a blueprint for future large-scale water resource monitoring.

## Introduction

The ability to sustainably manage water resources to avoid long-term depletion or environmental harm is of increasing importance due to the numerous alarming observations of diminishing global freshwater resources^1–4^. Approximately 80% of the population worldwide resides in an area with present threats to water security^5^. Over 50% of the world’s largest aquifers show declining trends in groundwater storage^6^ and 1.7 billion people inhabit areas where groundwater resources are considered stressed^7^. Many of these water shortages and groundwater declines affect transboundary regions—such as the Middle East and India-Pakistan border region—which is particularly worrisome given the already tense political relations in these areas coupled with the lack of institutional transparency and international cooperation^8–10^. Within the United States, of greatest concern is the Southwest, which includes the transboundary Colorado River basin that shares a border with Mexico and contains seven US states. Changes in snowmelt are driving seasonal shifts in extremes^11^ and near-decadal groundwater and surface water declines have been reported in this region due to the high intensity of water resource management primarily for irrigation^12–14^.

The magnitude and extent of global water scarcity has been examined in a number of previous studies^15–20^. One critical outcome of these studies is the finding that nearly one-quarter of global river basins are observed to be under severe water stress by comparing water demand to availability^16^. In addition, findings indicate projected global blue (renewable surface and groundwater) and green water (precipitation that naturally infiltrates) shortages of 36% and 50%, respectively^17^. Using a different approach, monthly modeled and observed data were used to identify that nearly 40% of the global population inhabits river basins with blue water shortages^18^.

Collectively, this research has provided deeper insight into the global sustainability of water management systems, but several key deficiencies exist with the formulation and calculation of these indices. First, the use of different models to generate water scarcity estimates is responsible for inconsistencies among these studies, as the number of people reported as inhabiting regions with severe water stress differs by up to a factor of almost eight^20^. In some cases the estimates of water availability do not account for the total amount of water (surface water and groundwater) that is available to a given area due to, for example, inter-basin water transfer and deep groundwater storage, with some studies omitting groundwater resources from the analysis completely^5^. Given the large exchange of water taking place between groundwater and surface water in the hyporheic zone^21, 22^, failing to integrate both sources into these assessments likely results in underestimation of total water availability and thus overestimation of water stress. Finally, consideration of ecosystem requirements is often missing^23^.

**Figure 1:**
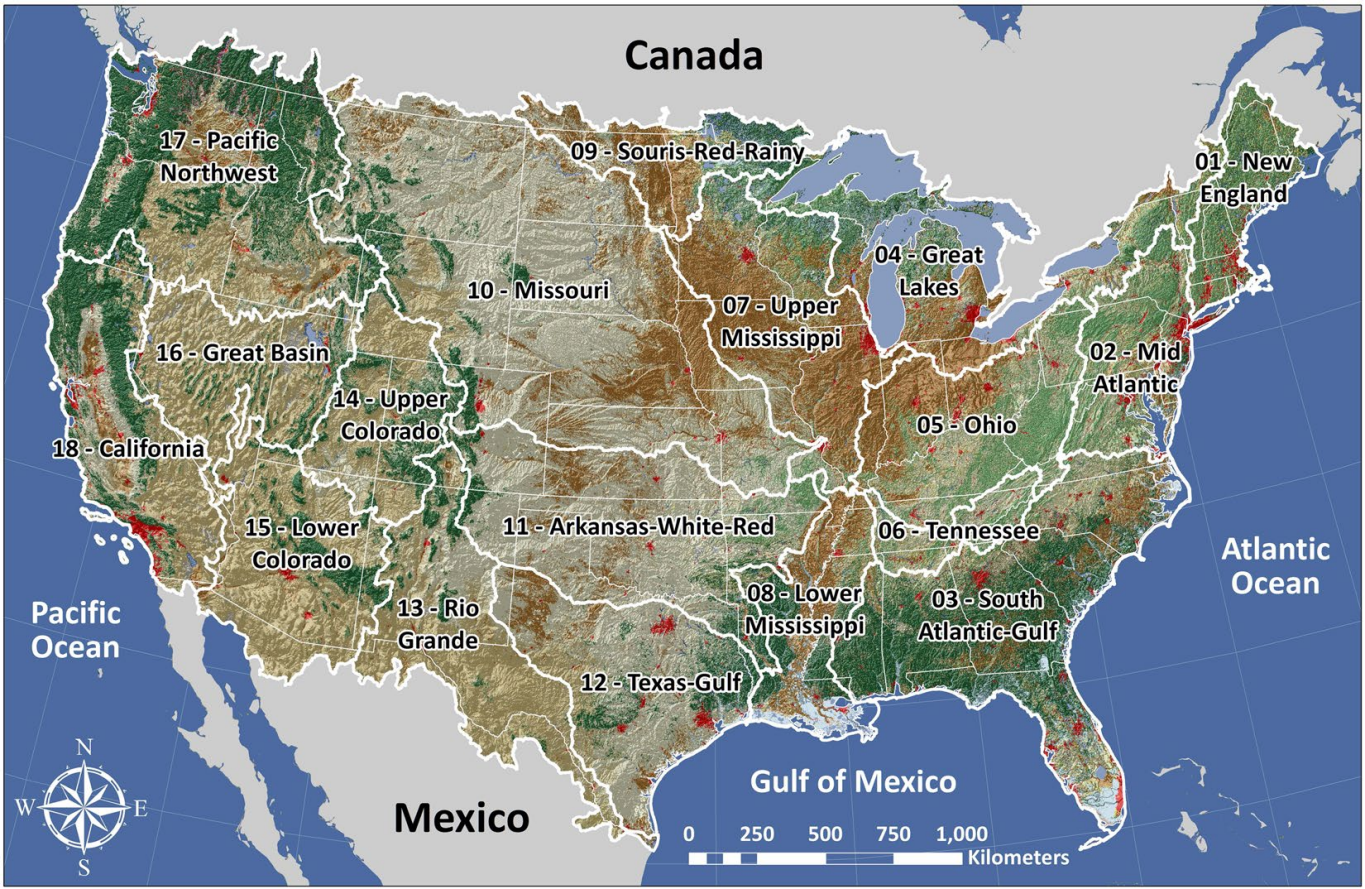
Map of 18 HUC 2-digit basins covering the contiguous US (Created using ArcGIS 10.4: http://desktop.arcgis.com/en/arcmap/10.4/get-started/main/get-started-with-arcmap.htm)^26^.

**Figure 2:**
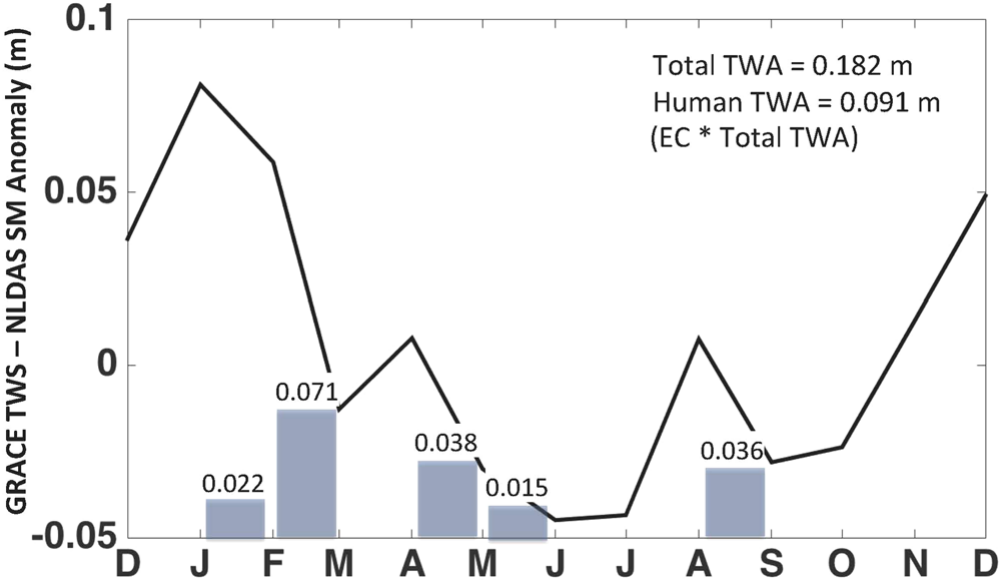
Conceptual diagram demonstrating how to calculate annual TWA. The black line represents the difference in monthly GRACE TWS and NLDAS soil moisture anomalies. The annual TWA is estimated as the cumulative decrease in month-to-month TWS and soil moisture differences (grey bars). The human TWA represents the portion of the total water available to humans after accounting for environmental demands using the environmental coefficient value, which is set to 0.5 in this example.

Here, we address these deficiencies by developing a comprehensive method that relies on satellite observations to compare total water availability (TWA), including all available aboveground and belowground water, to consumptive water use (water lost to evaporation as opposed to return flows to rivers and groundwater). NASA’s Gravity Recovery and Climate Experiment (GRACE) mission is used to extract the depth-integrated change in terrestrial water mass based on gravity measurements beneath the orbital path of the satellites^24^. This observation includes lumped changes in snow, surface water, soil moisture, and groundwater and has been used to estimate changes in total water storage^25^, or combined with other estimates to isolate changes in groundwater^6, 8, 10, 13, 14^. In order to calculate the water that is available to be harvested for human application, we remove a soil moisture estimate from the GRACE time series following previous approaches^8^ to arrive at the quantity known as “accessible water”^14^. This yields a time series estimate of snow, surface water and groundwater, which constitutes the gross human-available water in each of the 18 major HUC-2 watersheds (Fig. 1)^26^. TWA is then calculated by removing soil moisture from the sum of month-to-month decreases in the human-available groundwater and surface water estimate every year (Fig. 2), while accounting for consumptive water use and ecosystem requirements (see Methods).

Heightened concerns over unsustainable water management practices, in part, have given rise to methods that quantify environmental flow thresholds (EFTs) to account for environmental demands and prevent aquatic ecosystem collapse^23^. EFTs that are currently in use collectively range from 20–77% of mean runoff and/or groundwater—that is, the amount of water that can be removed without negatively impacting the ecosystem—considered at either daily, monthly, or annual timescales^21, 27–29^. Here, environmental demands are accounted for using the spatially distributed environmental water requirements (EWRs)^21^, which is referred to as the environmental coefficient (EC). Consumptive water use is calculated by estimating the consumptive portion of United States Geological Survey (USGS) water use records using an irrigation efficiency value of 0.6^18, 30–33^. This is done to acknowledge that some of the water reported in the USGS as water use inevitably gets recycled back to the ground and is thus still contributing to total water availability and not consumed. For example, water that is applied to crops will either infiltrate into the ground as groundwater or be incorporated into surface water runoff. Both the EC and irrigation efficiencies were allowed to vary by ±0.15 (a standard sigma range of uncertainty) as these estimates have never been measured across large space and time scales and actual water stress could be more severe (or conservative) than our reported mean values. Uncertainty in GRACE is very small in comparison to the 15% uncertainty for EC and irrigation efficiencies that were applied to the final estimate. Moreover, the uncertainty in monthly GRACE estimates (typically +/−0.5 cm for basins of this size)^25^ get quite small when propagated into a trend and are thus are not included in the overall uncertainty shown in this study.

**Figure 3:**
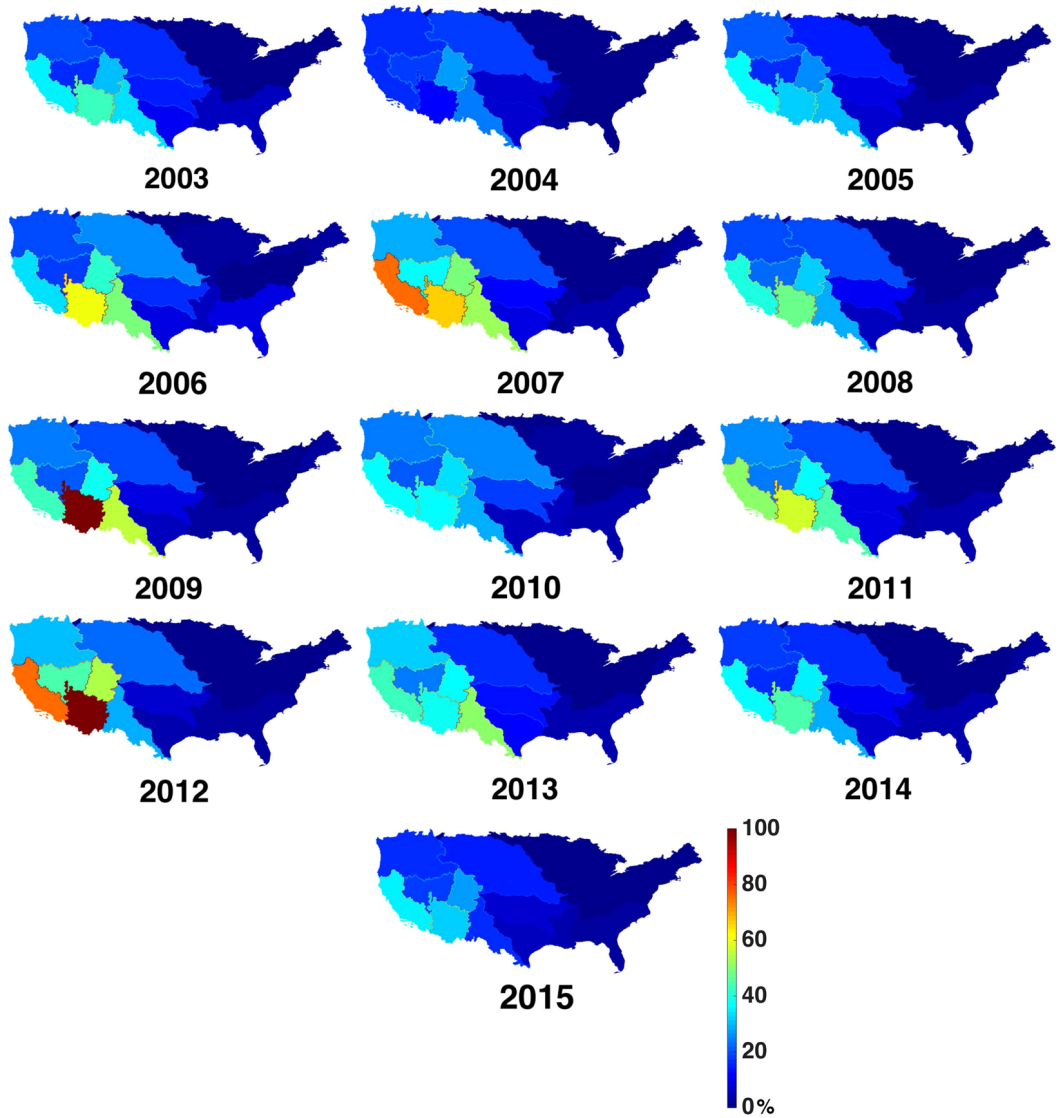
Annual 2003–2015 percent consumptive water use to availability ratios for the 18 two-digit HUC regions of the continental United States. Values shown represent results obtained when median environmental coefficient and irrigation efficiency parameters were used (created using Matlab version 2015b: https://www.mathworks.com/products/new_products/release2015b.html).

**Figure 4:**
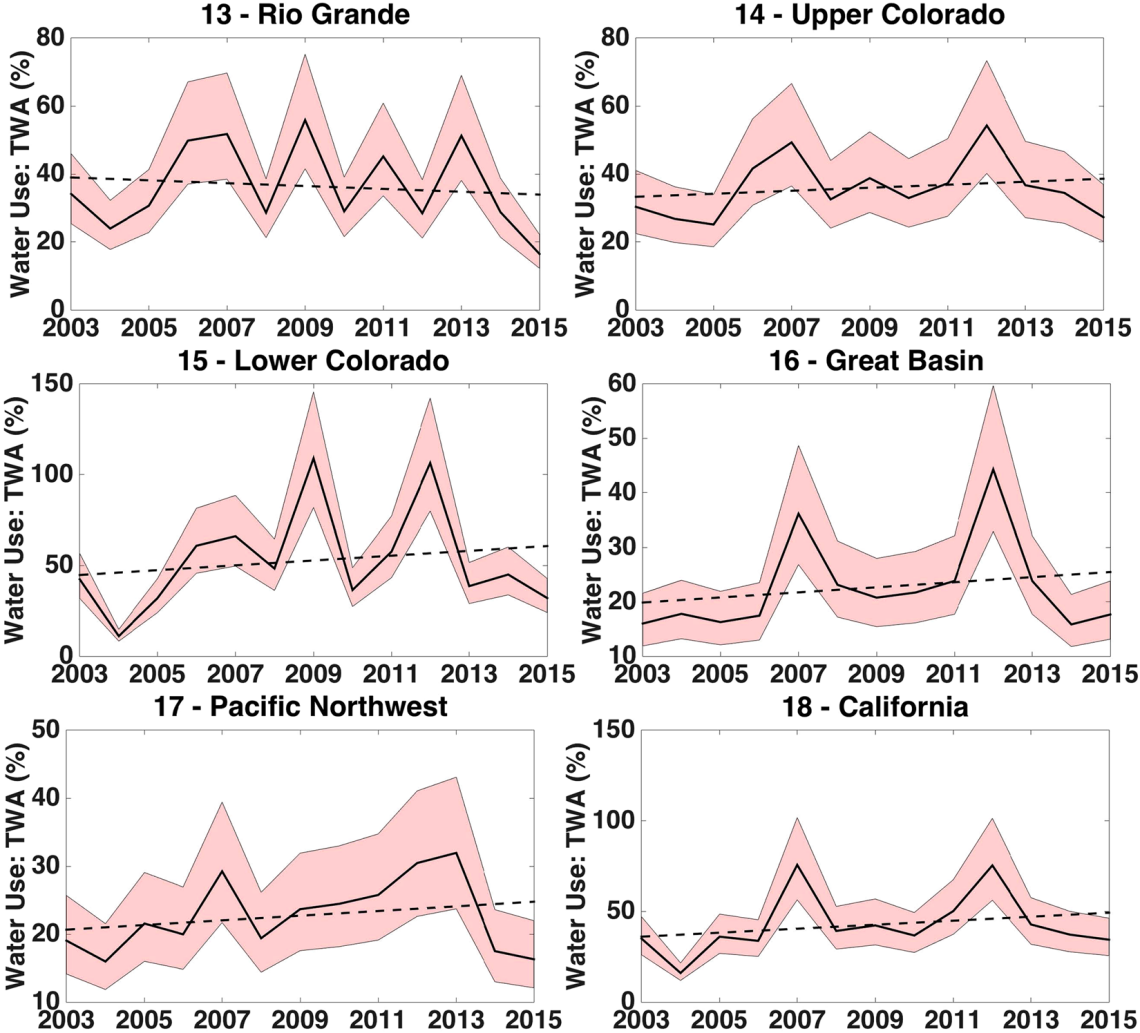
2003–2015 time series of percent consumptive water use to availability ratios for the southwest two-digit HUC regions. Solid black line represents results obtained when median environmental coefficient of 0.5 and water efficiency ratio of 0.6 were used. Associated linear trends (dashed black line) in water use to availability fractions are as follows: (−0.42% yr^−1^, p-value = 0.67), 14 Upper Colorado (0.44% yr^−1^, p-value = 0.51), 15 Lower Colorado (1.33% yr^−1^, p-value = 0.55), 16 Great Basin (0.47% yr^−1^, p-value = 0.48), 17 Pacific Northwest (0.34% yr^−1^, p-value = 0.41), and 18 California (1.10% yr^−1^, p-value = 0.39). Shaded regions indicate possible estimates given full range of parameter values.

The results from our work indicate that within the United States (US), the highest degree of water scarcity during 2003–2015 occurred in the Southwest (Fig. 3). Use of the median parameter values for the EC and irrigation efficiencies show consumptive water use exceeded 100% annual TWA twice in the Lower Colorado, which is largely made up of Arizona. In addition, the 75% threshold was surpassed twice in California and the Rio Grande reached a high mark of 56% in 2009, which are still indicative of water resource exploitation^34^. Overall mean consumptive water use (2003–2015) to availability ratios in the west ranged from a low of 23% in the Great Basin to a high of 53% in the Lower Colorado. Figure 4 shows the entire range of use to availability ratios over the study period for the six western basins resulting from the different parameter value combinations. Five of the six basins had an increasing trend of use to TWA. Overall ratios ranged from –0.42% yr^−1^ in the Rio Grande to 1.33% yr^−1^ in the Lower Colorado—though none of the trends were significant at the 95%-level. Given that no observations exist to truly validate these estimates and that they are predominately based on observations from GRACE and reported water use, there is no reason to hypothesize that these estimates would be erroneous and validations from previous modeled work are not shown.

Given the uncertainty in EC and irrigation efficiency parameter values, the analysis was also run using more extreme parameter values. Use of the most extreme values resulted in annual consumptive water use exceeding availability in both the Rio Grande and Lower Colorado HUC-2 basins. Preventing use from exceeding availability under this scenario would require water use curtailments in these basins of 14% and 43%, respectively.

Unsustainable water use practices have dire consequences for aquatic ecosystems and signs suggest that some of these impacts might already be occurring. For instance, an environmental minimum flow threshold of 10% is reported to be necessary for maximum ecological protection to prevent any deleterious effects on aquatic ecosystems^34^. Adhering to this standard in this study, however, would cause mean consumptive water use to exceed water availability in all six western HUC regions even when the most conservative irrigation efficiency estimate is applied. Furthermore, evidence indicates that the looser environmental standards are causing major ecological impairments both globally and in the southwestern US. For example, a review of global river flow alteration studies revealed 92% of 165 studies showed consistently negative changes occurring in fish species abundance, diversity, and demographic parameters due to these hydrological modifications^35^. Similarly, from 1979–2008, the number of vulnerable, threatened, endangered, and extinct North American freshwater species steadily rose^36^. Within the Southwest US alone, even slight flow alterations were observed to favor the establishment of invasive vegetation in riparian areas over native species^37^. In California, freshwater fish species that are extinct or at-risk of extinction rose from 62% in 1989 to 83% in 2011. Major flow augmentations were listed as one of the main causes of this population shift^38^.

Contrary to previous studies, our methods rely more on observations from *in-situ* water use data and satellite-based total water storage (TWS) obtained from GRACE. We note here that water stress has been quantified using observations from GRACE^6^, but that study focused solely on groundwater and based TWA estimates on the annual trend in groundwater storage changes rather than the approximate TWS amplitude as is done here.

Our framework provides an excellent opportunity to explore the sustainability of water resource management systems for groundwater and surface water combined, including identifying regions where intervention is required. The use-to-availability levels we calculated in the southwestern US are indicative of unsustainable water resource management practices^39^. The level of irrigation water use (which has a higher water consumption, see Methods) relative to water availability in the Southwest suggests similar findings for other agriculturally intense regions located in semi-arid climates across the globe. Still, uncertainty and lack in robust or reported water use data worldwide may pose additional constraints in conducting this analysis in other regions, especially within developing countries. The study also offers a foundation for further investigations of sustainable water use from remote sensing at different spatial and temporal scales, incorporating more rigorous validation estimates when better estimates of evapotranspiration (ET) become available to close the water budget, and more robust estimates of environmental water requirements. Given the success of satellite missions with global observations in hydrology^40^, use of remote sensing to determine water stress will allow for robust, global evaluations including geopolitically sensitive regions where *in-situ* observations and data sharing are lacking, while increasing the consistency in results among future studies. Such efforts would be beneficial towards better pinpointing areas where immediate changes to water management practices are necessary to mitigate water stress and avoid major threats to society and the environment.

## Methods

Data used to calculate TWS came from the Gravity Recovery and Climate Experiment (GRACE) satellite mission^24^. GRACE is used to examine global monthly changes in the gravity field over large areas (>200,000 km^2^), which are directly related to the TWS changes at the spatial and temporal resolution. Grid scaling factors were applied to remove leakage effects from sampling and post-processing of GRACE observations. Monthly gaps were filled using linear interpolation. This technique was applied under the assumption that monthly GRACE observations are typically not consecutive, nor do they exist at peak monthly maxima or minima storage. As such, use of linear interpolation to fill in missing months makes little difference in the results. Soil moisture data over the same period was derived using a multi-model mean from the VIC, Noah, and Mosaic land surface models running within the North American Land Data Assimilation System (NLDAS)^41^.

Water use data comes from the United States Geological Survey (USGS) National Water-Use Information Program, which collects comprehensive, county-level surface water and groundwater use statistics across all sectors every five years. The data is available for download directly from the USGS website (http://water.usgs.gov/watuse/). Here, we use the 2005 and 2010 data to coincide with the GRACE observation period^42, 43^. Because only two years of data were available, we use a benchmark average to represent water use over the entire study period. Although irrigation accounts for 80–90% of consumptive water use overall^20, 31–33^, industrial and domestic water use contributes a much greater proportion in more urbanized areas so these quantities were also included in the total water use estimates.

The GRACE, NLDAS, and USGS data were scaled to the two-digit Hydrologic Unit Code (HUC) USGS watershed boundaries (Fig. 1)^34^ due to the coarse spatial resolution of GRACE. As watersheds represent the spatial unit most commonly associated with water resource management, distributing the data at this scale provides a practical approach to presenting water use with respect to availability across the landscape. County-level data from the USGS and grid-based data from GRACE that were intersected by a HUC basin divide were dealt with by weighting the data in direct proportion to the area that fell on either side of the boundary. The analyses conducted for this study focused only on basins from the continental United States.

Annual TWA for the 18 two-digit HUC basins was calculated using a new method shown in Equation 1:

1 $$TWA({t}_{yr})=\sum _{t=-1}^{12}[(TWS({t}_{mo})-TWS({t}_{mo}+1))-(SM({t}_{mo})-SM({t}_{mo}+1))]\ast EC$$

$$TWS({t}_{mo}) > TWS({t}_{mo}+1)$$

$$SM({t}_{mo}) > SM({t}_{mo}+1)$$

where TWA(t_yr_) is the annual total water availability (m^3^ yr^−1^), TWS(t_mo_) is the monthly total water storage change from GRACE (m^3^ mo^−1^), SM(t_mo_) is the monthly soil moisture storage change from NLDAS (m^3^ mo^−1^), and EC is the environmental coefficient (Fig. 2), which was added to account for environmental demands. The notation (t_mo_ + 1) refers to the next month and the analysis begins in December from the previous year (noted as t = −1) and goes through December of the current year (noted as t = 12), as shown in Fig. 2. Here, we use the two-digit HUC basin EC values^23^, which were also based on groundwater recharge and changes in surface water storage, as estimated from a global hydrology model. Note that in Equation 1, the constraints indicate that the cumulative difference in month-to-month TWS and SM was calculated by summing the TWS and SM differences for each year starting on December of the previous year only for consecutive months where the TWS and SM of the trailing month exceeded that of the lead month. This is done to account for all the water that might be available for use, as more than one TWA maximum might occur throughout the year depending on the climate. The volume represented by SM is necessarily subtracted from TWS, as this amount is not considered to be available for use. Both the GRACE and NLDAS data are presented in the form of anomalies relative to the 2004–2009 period.

The general principle behind Equation 1 is that within each year, the TWS reaches a maximum in a given basin largely due to seasonally elevated precipitation (inflows) that exceeds ET (outflows), leading to an accumulation of surface water and groundwater within the basin. Correspondingly, TWS falls to an annual minimum when the levels of ET exceed precipitation, resulting in more water being “consumed” and leaving the basin through the atmosphere. Thus, the accumulation of water losses (both surface water and groundwater) that occur between these two thresholds within the basin derived from GRACE can be taken as the total “active” amount of available water for use from the system within a given year. When the total use of water from humans and environment exceeds this amount, less water will be available in the following year, resulting in unsustainable water use.

In this approach, we remove changes in naturally occurring soil moisture derived from NLDAS from this estimate because although soil moisture is used by some aspects of the environment (e.g. plants and bacteria), it is not physically being removed from the surface or underground by humans in the case of rain-fed agriculture. Therefore, it is deemed inaccessible to humans and thus including it would lead to an overestimation of TWA. Contrastingly, increases in soil moisture from irrigation are counted as water use and lead to a higher consumptive water use to availability ratio. In addition, we assume all snow water storage to be melted and thereby available for use because we are ultimately only interested in estimating the water occurring within a given year that is or will be available for use so the method described here was deemed appropriate. Likewise, we did not attempt to explicitly include changes in river discharge into the analysis (in addition to what we derive from GRACE and NLDAS), as this would result in double-counting some of the contributions from snowmelt, resulting in an over-estimation of total water availability.

Basin-distributed environmental coefficients are applied to account for the portion of the resulting volume that must remain in the environment to minimize damage to the ecosystem. Hence, it is assumed the residual water must stay in the environment in the form of stored surface water and groundwater to be available for environmental use. Although these were originally developed to determine environmental water requirements for rivers over large basins, we apply them in this study to all surface water and groundwater because of the dependence of the environment on these other water sources. Moreover, the strong interconnectedness of groundwater and other surface water forms to rivers provides justification for application of the same environmental water requirements in this study^22^. As also stated in their original application, we acknowledge that the environmental coefficients we use should be interpreted as “rules-of-thumb” for incorporating environmental water demands into large-scale studies of sustainable water use. Finer scale studies warrant more detailed assessments of how different levels of groundwater and surface water use impact environmental water availability over different times of the year^21^.

Annual consumptive water use was calculated using Equation 2:

2 $$CWU({t}_{yr})={U}_{irr}\ast CF+{U}_{dom}\ast 0.20+{U}_{ind}\ast 0.15$$

where U_irr_ is the irrigation (m^3^), U_dom_ is the domestic (m^3^), and U_ind_ is the industrial annual water use (m^3^). CF is the consumptive water use fraction of the irrigation water use, otherwise known as the irrigation efficiency and is assumed to be 60%^20, 31–34^. Note that we assume the consumptive water use fraction of domestic and industrial water use to be 20% and 15%, respectively. Annual consumptive use from Equation 2 was compared to TWA from Equation 1 for each HUC basin using Equation 3:

3 $$CWU:TW{A}_{an}=100-\frac{[TWA({t}_{yr})-CWU({t}_{yr})]}{TWA({t}_{yr})}\,\ast 100$$

where CWU:TWA_an_ is the annual consumptive water use to total water availability ratio (%) and is identical to the Water Stress Indicator (WSI)^20^.

Given the known uncertainty in the environmental coefficient and irrigation efficiency parameter values, a parameter sensitivity analysis in Supplementary Table 1 was used to present the mean annual consumptive water use to availability ratios calculated using Equation 3. Both parameters were allowed to vary by +/−0.15 to assume a standard sigma range. Parameter sensitivity results were shown only for the six western HUC basins due to preliminary tests that revealed the ratios to be highest in this region.

### Data availability

This study used GRACE Release 05 data, which were generated from the Center for Space Research, University of Texas at Austin and are available from the NASA Jet Propulsion Laboratory Physical Oceanography Distributed Active Archive Center (http://podaac.jpl.nasa.gov). Water use data from the United States Geologic Survey (USGS) originated from the National Water-Use Information Program, which is available for download directly from the USGS website (http://water.usgs.gov/watuse/). Soil moisture data from the National Land Data Assimilation System (NLDAS) was derived using the mean of the NLDAS-2 VIC, Noah, and Mosaic Land Surface Model data products, which were acquired as part of the mission of NASA’s Earth Science Division and archived and distributed by the Goddard Earth Sciences (GES) Data and Information Services Center (DISC). This data is available for download at the NASA Land Data Assimilation Systems website (http://ldas.gsfc.nasa.gov/nldas/NLDAS2model_download.php).

## Electronic supplementary material

Supplementary Information

## References

[1] Vörösmarty CJ, Green P, Salisbury J, Lammers RB (2000). Global Water Resources: Vulnerability from climate change and population growth. Science.

[2] Oki T, Kanae S (2006). Global hydrological cycles and world water resources. Science.

[3] Alcamo J, Flörke M, Märker M (2007). Future long-term changes in global water resources driven by socio-economic and climatic changes. Hydrol. Sci. J..

[4] Schewe J (2014). Multimodel assessment of water scarcity under climate change. Proc. Natl. Acad. Sci..

[5] Vörösmarty CJ (2010). Global threats to human water security and river biodiversity. Nature.

[6] Richey AS (2015). Quantifying renewable groundwater stress with GRACE. Water Resour. Res..

[7] Gleeson T, Wada Y, Bierkens MFP, van Beek LPH (2012). Water balance of global aquifers revealed by groundwater footprint. Nature.

[8] Rodell M, Velicogna I, Famiglietti JS (2009). Satellite-based estimates of groundwater depletion in India. Nature.

[9] Biancamaria S, Hossain F, Lettenmaier DP (2011). Forecasting transboundary river water elevations from space. Geophys. Res. Lett..

[10] Voss KA (2013). Groundwater depletion in the Middle East from GRACE with implications for transboundary water management in the Tigris-Euphrates-Western Iran Region. Water Resour. Res..

[11] Solander, K. C., Bennett, K. E. & Middleton, R. S. Shifts in historical streamflow extremes in the Colorado River Basin. *J. Hydrol: Reg. Studies*, **12**, 363–377 (2017).

[12] Christensen NS, Lettenmaier DP (2007). A multimodel ensemble approach to assessment of climate change impacts on the hydrology and water resources of the Colorado River Basin. Hydrol. Earth Syst. Sci..

[13] Famiglietti JS (2011). Satellites measure recent rates of groundwater depletion in California’s Central Valley. Geophys. Res. Lett..

[14] Castle SL (2014). Groundwater depletion during drought threatens future water security of the Colorado River Basin. Geophys. Res. Lett..

[15] Falkenmark M (1989). The massive water scarcity now threatening Africa: Why isn’t it being addressed?. Ambio.

[16] Alcamo J (2003). Global estimates of water withdrawals and availability under current and future “business-as- usual” conditions. Hydrol. Sci. J..

[17] Rockström J (2009). Future water availability for global food production: the potential of green water for increasing resilience to global change. Water Resour. Res..

[18] Hoekstra AY, Mekonnen MM, Chapagain AK, Mathews RE, Richter BD (2012). Global monthly water scarcity: blue water footprints versus blue water availability. PLoS ONE.

[19] Wada Y, Bierkens MFP (2014). Sustainability of global water use: past reconstruction and future projections. Env. Res. Lett..

[20] Wada Y (2011). Global monthly water stress: 2. Water demand and severity of water stress. Water Resour. Res..

[21] Smakhtin V, Revenga C, Döll P (2004). A pilot global assessment of environmental water requirements and scarcity. Water Int..

[22] Findlay S (1995). Importance of surface-subsurface exchange in stream ecosystems: The hyporheic zone. Limnol. Oceanogr..

[23] Richter BD, Baumgartner JV, Powell J, Braun DP (1996). A method for assessing hydrologic alteration within ecosystems. Conserv. Biol..

[24] Tapley BD, Bettadpur S, Ries JC, Thompson PF, Watkins MM (2004). GRACE Measurements of mass variability in the Earth System. Science.

[25] Scanlon BR (2017). Global evaluation of new GRACE mascon products for hydrologic applications. Water Resour. Res..

[26] Seaber PR, Kapinos FP, Knapp GL (1987). Hydrologic Unit Maps: U.S. Geological Survey. Water-Supply Paper.

[27] Hanasaki N (2008). An integrated model for the assessment of global water resources – Part 1: Model description and input meteorological forcing. Hydrol. Earth Syst. Sci..

[28] Tenant D (1976). Instream flow regimens for fish, wildlife, recreation and related environmental resources. Fisheries.

[29] Cullen, P. The future of flow restoration in Australia. *WaterShed*, September: 1–2 (2001).

[30] Döll P, Siebert S (2002). Global modeling of irrigation water requirements. Water Resour. Res..

[31] Rost S (2008). Agricultural green and blue water consumption and its influence on the global water system. Water Resour. Res..

[32] FAO, Irrigation water requirement and water withdrawal by country. K. Frenken and V. Gillet (authors), *Food and Agriculture Organization of the United Nations*, Rome, Italy, 263 p. (2012).

[33] Döll P, Schmied HM, Schuh C, Portmann FT, Eicker A (2014). Global-scale assessment of groundwater depletion and related groundwater abstractions: combining hydrological modeling with information from well observations and GRACE satellites. Water Resour. Res..

[34] Richter BD, Davis MM, Apse C, Konrad C (2011). SHORT COMMUNICATION: A Presumptive Standard for environmental flow protection. Riv. Res. Appl..

[35] Poff NL, Zimmerman JKH (2010). Ecological responses to altered flow regimes: a literature review to inform the science and management of environmental flows. Freshwater Biol..

[36] Jelks HL (2008). Conservation status of imperiled North American freshwater and diadromous fishes. Fisheries.

[37] Merrit DM, Poff NL (2010). Shifting dominance of riparian Populus and Tamarix along gradients of flow alteration in western North American rivers. Ecol. Appl..

[38] Moyle PB, Katz JVE, Quiñones RM (2011). Rapid decline of California’s native inland fishes: A status assessment. Biol. Conserv..

[39] Smakhtin, V., Revenga, C. & Döll, P. Taking into account environmental water requirements in global-scale water resources assessments. Comprehensive Assessment Research Rep. 2, Comprehensive Assessment Secretariat, Colombo, Sri Lanka 22 (2004).

[40] Famiglietti JS (2015). Satellites provide the big picture. Science.

[41] Mitchell KE (2004). The multi-institution North American Land Data Assimilation System (NLDAS): Utilizing multiple GCIP products and partners in a continental distribtuted hydrological modeling system. J. Geophys. Res..

[42] Kenny JF (2005). Estimated use of water in the United States in 2005. US Geological Survey Circular.

[43] Maupin MA (2014). Estimated use of water in the United States in 2010. US Geological Survey Circular.

