# Supplementary material for: GRACE satellite observations reveal the severity of recent water over-consumption in the United States
Source: Sci Rep. 2017 Aug 18;7:8723. doi: 10.1038/s41598-017-07450-y (PMC5562833; doi:10.1038/s41598-017-07450-y)
Supplement: Supplementary file 1 — Supplementary Information [file 41598_2017_7450_MOESM1_ESM.pdf]

GRACE satellite observations reveal the severity of recent water over-consumption in the United States

Kurt C. Solander[a*], John T. Reager[b], Yoshihide Wada[c,d,e], James S. Famiglietti[b], Richard S. Middleton[a]

[a]Los Alamos National Laboratory, Los Alamos, NM 87545, USA

[b]Jet Propulsion Laboratory, California Institute of Technology, 4800 Oak Grove Drive, Pasadena, California 91009, USA

[c]International Institute for Applied Systems Analysis, Laxenburg, Austria

[d]Center for Climate Systems Research/NASA Goddard Institute for Space Studies, Columbia University, 2880 Broadway, New York, New York 10025, USA

[e]Department of Physical Geography, Utrecht University, The Netherlands

*Corresponding author: ksolander@lanl.gov

Supplementary Information

The parameter sensitivity results in Supplementary Table 1 indicate higher sensitivity to the irrigation water use efficiencies than the environmental coefficient, as the magnitude change in results was greater for each incremental change in the irrigation efficiencies. The magnitude of change in the ratios is also lower for higher environmental coefficient values, while negligible differences were observed among the ratio differences for shifts in low versus high irrigation water use efficiencies.

Supplementary Table 2 presents national water balance results based on the median, maximum, and minimum parameter values. The annual percent water use to availability ratios were calculated by weighting the fractions according to HUC basin size. Therefore, the first column of information provides an indication of the overall spatial coverage of this result on an annual basis, while the actual balance of use to availability is presented in the final column. Water use to availability ratios over the continental United States ranged from a low of 10% in 2004 to a high of 22% in 2012. Extreme values for the balance of TWA and water use ranged from a low of approximately 400 billion m^3 in 2006 to a high of 860 billion m^3 in 2004. The mean, maximum, minimum, and standard deviation shown in this table are based on those determined from each HUC basin over the study period and are therefore much higher or lower than implied by the 2003-2015 annual data.

Figure S1 shows the 2003-2015 mean, maximum, minimum, and standard deviation of benchmark consumptive water use to availability fractions resulting from the median set of parameter values. Focusing on the west, consumptive water use exceeded 11-25% water availability when the minimum TWA was used and 32-109% when the maximum TWA was

used. The plot displaying the maximum TWA is strikingly analogous to the 2012 plot from Figure 2, as the 75% consumption to availability ratio also occurred in the two southwestern HUC regions during this time. Overall, the standard deviation was much higher in the southwestern basins, pointing to the larger degree of water resource variability in this region relative to elsewhere in the United States.

Supplementary Table 1. Sensitivity analysis of irrigation efficiency and environmental coefficient affects on mean 2003-2015 consumptive water use to availability.

**13 – Rio Grande**			**Irrigation Efficiency (%)**				
**Env Coefficient**	**45**	**50**	**55**	**60**	**65**	**70**	**75**
**0.57**	35	39	42	46	50	53	57
**0.62**	32	36	39	42	46	49	53
**0.67**	30	33	36	39	42	45	49
**0.72**	28	31	34	37	39	42	46
**0.77**	26	29	31	34	37	40	42
**0.82**	24	27	29	32	35	37	40
**0.87**	23	25	28	30	33	35	38
**14 – Up Colorado**			**Irrigation Efficiency (%)**				
**Env Coefficient**	**45**	**50**	**55**	**60**	**65**	**70**	**75**
**0.48**	35	39	43	47	51	55	59
**0.53**	32	36	39	43	46	50	53
**0.58**	29	33	36	39	42	46	49
**0.63**	27	30	33	36	39	42	45
**0.68**	25	28	31	33	36	39	42
**0.73**	23	26	28	31	34	36	39
**0.78**	22	24	27	29	31	34	36
**15 – Low Colorado**			**Irrigation Efficiency (%)**				
**Env Coefficient**	**45**	**50**	**55**	**60**	**65**	**70**	**75**
**0.48**	54	59	64	69	75	80	85
**0.53**	49	53	58	63	67	72	77
**0.58**	44	49	53	57	62	66	70
**0.63**	41	45	49	53	57	61	65
**0.68**	38	42	45	49	53	56	60
**0.73**	35	39	42	46	49	52	56
**0.78**	33	36	39	43	46	49	52
**16 – Great Basin**			**Irrigation Efficiency (%)**				
**Env Coefficient**	**45**	**50**	**55**	**60**	**65**	**70**	**75**
**0.58**	22	24	26	29	31	33	35
**0.63**	20	22	24	26	28	31	33
**0.68**	18	20	22	24	26	28	30
**0.73**	17	19	21	23	25	26	28
**0.78**	16	18	20	21	23	25	26
**0.83**	15	17	18	20	22	23	25
**0.88**	14	16	17	19	20	22	23
**17 – Pac Northwest**			**Irrigation Efficiency (%)**				
**Env Coefficient**	**45**	**50**	**55**	**60**	**65**	**70**	**75**
**0.52**	22	25	27	29	32	34	36
**0.57**	20	22	25	27	29	31	33
**0.62**	19	21	23	25	27	29	31
**0.67**	17	19	21	23	25	26	28
**0.72**	16	18	19	21	23	25	26
**0.77**	15	17	18	20	21	23	25
**0.82**	14	16	17	19	20	22	23
**18 - California**			**Irrigation Efficiency (%)**				
**Env Coefficient**	**45**	**50**	**55**	**60**	**65**	**70**	**75**
**0.62**	40	45	49	53	57	61	66
**0.67**	37	41	45	49	53	57	61
**0.72**	35	38	42	46	49	53	57
**0.77**	33	36	39	43	46	49	53
**0.82**	31	34	37	40	43	46	50
**0.87**	29	32	35	38	41	44	47
**0.92**	27	30	33	36	39	41	44

Supplementary Table 2. Summary water balance statistics over the continental United States [a].

Year	Consumptive Water Use: TWA Ratio (%)[b]	TWA - Consumptive Water Use (m^3)
2003	14 [9 - 23]	5.1E+11 [3.5E+11 – 6.7E+11]
2004	10 [6 - 16]	8.6E+11 [6.2E+11 – 1.1E+12]
2005	12 [8 - 20]	6.6E+11 [4.6E+11 – 8.6E+11]
2006	18 [11 - 29]	4.0E+11 [2.7E+11 – 5.4E+11]
2007	21[13 - 33]	4.3E+11 [2.8E+11 – 5.7E+11]
2008	14 [9 - 23]	5.8E+11 [4.0E+11 – 7.6E+11]
2009	19 [12 - 30]	6.2E+11 [4.3E+11 – 8.1E+11]
2010	16 [10 - 26]	4.5E+11 [3.0E+11 – 6.0E+11]
2011	17 [11 - 27]	5.5E+11 [3.7E+11 – 7.2E+11]
2012	22 [14 - 35]	6.1E+11 [4.2E+11 – 8.0E+11]
2013	17 [10 - 27]	4.9E+11 [3.3E+11 – 6.6E+11]
2014	13 [8 - 22]	6.0E+11 [4.1E+11 – 7.8E+11]
2015	11 [7 - 18]	6.9E+11 [4.8E+11 – 8.9E+11]
MEAN[c]	16 [10 - 25]	5.6E+11 [3.9E+11 – 7.4E+11]
MAX[c]	26 [16 - 42]	9.7E+11 [7.0E+11 – 1.2E+12]
MIN[c]	9 [5 - 14]	2.9E+11 [1.8E+11 – 4.0E+11]
STD[c]	5 [3 - 8]	1.1E+10 [8.6E+09 – 1.4E+10]

[a] results based on median parameter values, while range in brackets calculated using least and most conservative parameter values
[b] weighted by two-digit HUC areas
[c] based on within-HUC-region results over study period

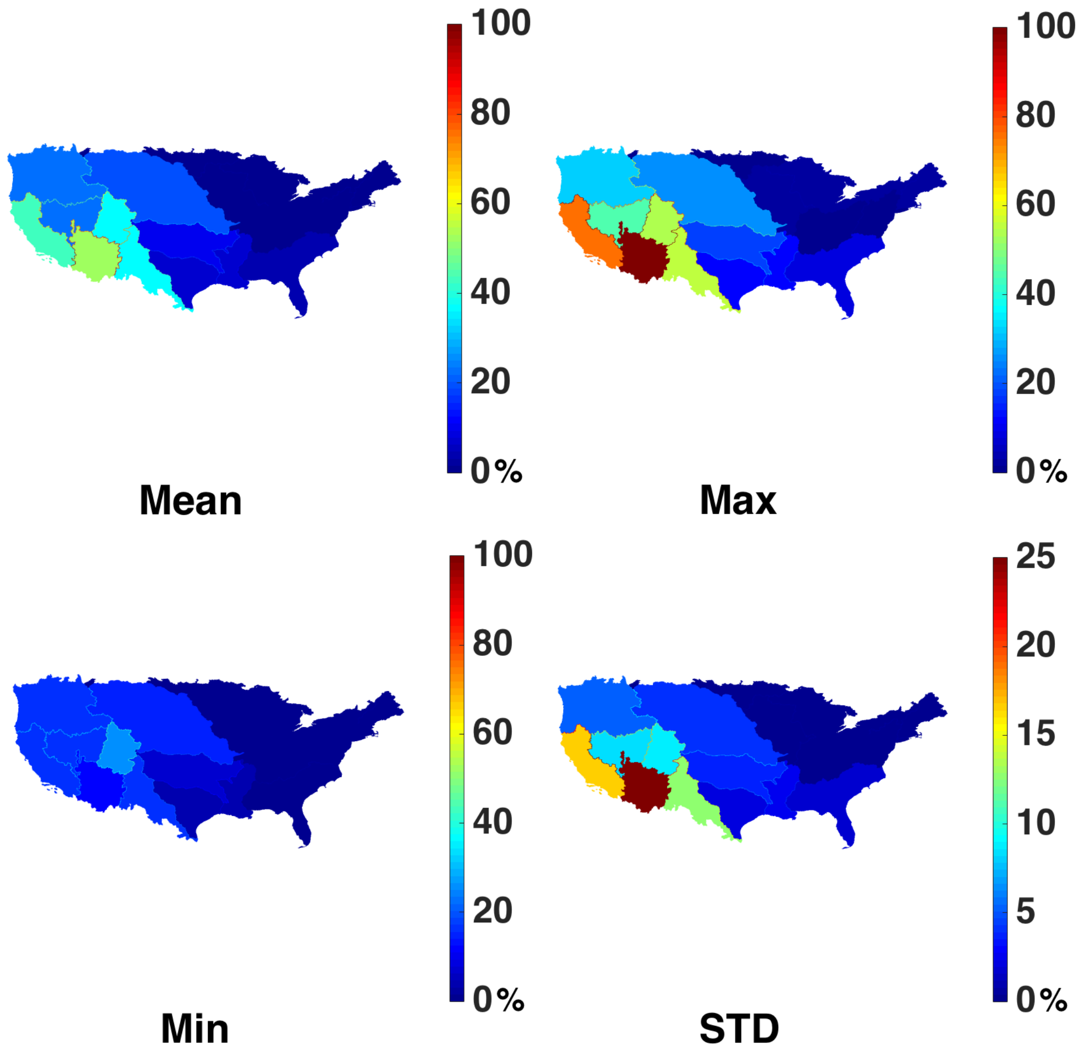

Supplementary Figure 1: 2003-2015 mean (top-left), maximum (top-right), minimum (bottom-left), and standard deviation (bottom-right) percent consumptive water use to availability ratios for the 18 two-digit HUC regions of the continental United States. Values shown represent results obtained when median environmental coefficient and irrigation efficiency parameters were used (created using Matlab version 2015b: https://www.mathworks.com/products/new_products/release2015b.html).
